# Environmental pH and compound structure affect the activity of short-chain carboxylic acids against planktonic growth, biofilm formation, and eradication of the food pathogen *Salmonella enterica*

**DOI:** 10.1128/spectrum.01658-24

**Published:** 2024-09-16

**Authors:** Ker-Sin Ng, Maria Florencia Bambace, Emilie Balleby Andersen, Rikke Louise Meyer, Clarissa Schwab

**Affiliations:** 1 Department of Biological and Chemical Engineering, Aarhus University, Aarhus C, Denmark; 2 Interdisciplinary Nanoscience Center, Aarhus University, Aarhus C, Denmark; 3 Department of Biology, Aarhus University, Aarhus C, Denmark; University of Minnesota Twin Cities, St. Paul, Minnesota, USA

**Keywords:** short-chain carboxylic acids, environmental pH, compound structure, hydrophobicity (log *K*_ow_), antimicrobial, biofilm, *Salmonella*

## Abstract

Short-chain carboxylic acids (SCCAs) that are naturally produced by microbial fermentation play an essential role in delaying microbial spoilage. SCCAs are structurally diverse, but only a few of them are routinely used in food biopreservation. This study investigated the effects of environmental pH and intrinsic properties of 21 structurally different SCCAs on the antimicrobial and antibiofilm activity against *Salmonella enterica*. Inhibition of SCCA toward planktonic and biofilm growth of *S. enterica* was higher in an acidic environment (pH 4.5) that is common in fermented products, and for SCCA that possessed both a high acid dissociation strength (pK_a_) (>4.0) and a positive hydrophobicity [octanol/water partition coefficient (log *K*_ow_)]. Crotonic and caproic acids were identified as SCCAs with potential as biopreservatives even at near-neutral pH. SCCA with hydrophilic groups such as lactic acid did not inhibit *S. enterica* at concentrations up to 50 mM, while SCCA with benzene or methyl groups or a double bond prevented *S. enterica* growth and biofilm formation. Stimulation of biofilm formation was observed for formic, acetic, and propionic acid close to the minimum inhibitory concentration to reduce 50% of cell density (MIC_50_) of planktonic cells, and for citric and isocitric acid with an MIC_50_ of ≥50 mM. The presence of low concentrations of formic and propionic acids during biofilm formation conferred protection during eradication possibly due to a pre-adaptation effect, yet two consecutive acid treatments were successful in eradicating biofilms if the first acid treatment was two- to threefold of the MIC_50_.

**IMPORTANCE:**

This study provides a systematic comparison on the antimicrobial and antibiofilm activity of more than 20 structurally different SCCAs against a common food pathogen. We tested the antimicrobial activity at controlled pH and identified the structure-dependent antimicrobial effects of SCCA without the confounding influence of acidification. The combined effect of pK_a_ and log *K*_ow_ was identified as an important feature that should be considered when deciding for a specific SCCA in the application as antimicrobial. Our results imply that additional phenomena such as the use of SCCA as substrate and cellular pre-adaption effects have to be taken into consideration. We finally present a two-step treatment as an efficient approach to eradicate biofilms, which can be applied for the disinfection of contact surfaces and manufacturing equipment. Results obtained here can serve as guidelines for application of SCCA to avoid the growth of food pathogens and/or to develop biopreserved food systems.

## INTRODUCTION

Short-chain carboxylic acids (SCCAs) that are naturally produced by plants or microbial fermentation have been used to reduce the presence of food pathogens (1, 2). SCCAs have a carbon number of less than or equal to six (C ≤6) and possess at least one carboxyl group (—COOH) (3). In general, the antimicrobial activity of SCCA increases with higher acid dissociation strength (pK_a_) and lower pH due to a higher concentration of undissociated acids, which can diffuse across the cell phospholipid bilayer (4). SCCAs that are approved by the European Food Safety Authority and the US Food and Drug Administration for usage as food additives and preservatives to inhibit food pathogens and contaminants include acetic, propionic, lactic, benzoic, succinic, tartaric, malic, and citric acids.

Among the food pathogens, *Salmonella* is one of the major sources of food contamination and accounted for 70% (773 of 1,099) of foodborne illness outbreaks in Europe (5) and 20% (94 of 479) in the United States (6) in 2021. *Salmonella enteric*a subsp. *enterica* serovar Typhimurium and Enteritidis are the primary serovars that lead to foodborne illnesses among the 2,500 serovars being identified to date (7, 8). *Salmonella* infection leads to severe symptoms of gasteroenteritis such as bloody diarrhea, fever, and stomach cramps; around one-fifth of the patients were hospitalized. *Salmonella* contamination on meat and particularly on broilers, eggs, and dairy products imposes a risk. *Salmonella* usually attaches on food contact surfaces and manufacturing equipment as biofilm that contaminates pre-cooked foods (9). Biofilm growth is characterized by cell adhesion on biotic/abiotic surfaces and aggregation within a polysaccharide matrix.

SCCAs have been investigated as antimicrobial against *Salmonella* before. For example, 0.5% of acetic (87 mM) or citric (26 mM) acids reduced the numbers of *S*. Typhimurium ATCC 14028 in tahini (sesame paste) by 2–4 log CFU/mL within 28 days (10), while the synergistic effect of 1.4% citric acid (73 mM) and 0.3% acetic acid (52 mM) inhibited the same strain during storage of ready-to-eat salads at different temperatures (11). Cosansu and Ayhan (12) found that dipping chicken meats in lactic acid (1%: 134 mM, 3%: 402 mM) and acetic acid (1%: 175 mM, 2%: 350 mM) was effective in delaying *Salmonella* growth (12).

When *Salmonella* grew as biofilm, SCCAs combined with additional treatments were more effective in reducing cell counts compared to SCCA alone. Electrostatic spraying of 2% lactic acid (268 mM) and malic acid (149 mM) reduced the *S*. Typhimurium SD 10 on cantaloupe rind by 3.5 log CFU/disk (13). Lactic acid (0.5%–2%, 67–268 mM) with UV-C (5–10 min) reduced *S*. Enteritidis biofilms by 3–6 log CFU/cm^2^ on stainless steel and silicone rubber and by 2 log CFU/g on chicken skin (14). A combination of steam and lactic acid showed additional 0.2–2.0 log reductions of *S*. Typhimurium on polyvinyl chloride and stainless-steel surfaces compared to individual treatments (15).

Most of the studies that investigated SCCAs as antimicrobial and antibiofilm agents against *Salmonella* focused on acetic and lactic acids. With a pK_a_ of 3.46, lactic acid is considered a stronger acid but a weaker antimicrobial than acetic acid (pK_a_ = 4.76). However, SCCAs are structurally diverse with different side branches and unsaturated bonds. The presence of hydrophobic or hydrophilic side groups that affects the hydrophobicity [octanol/water partition coefficient log *K*_ow_] of SCCA is often ignored (16), even though the cell membrane is more permeable to hydrophobic molecules. Previous research on the antimicrobial mechanisms of SCCA often did not control for acidifying activity, which renders the disentanglement of concurrent pH modification and antimicrobial activity difficult.

Considering that parameters defining the antimicrobial activity of SCCAs have not fully resolved and that biofilm formation represents an additional serious threat to food safety, the aim of this study was to systematically evaluate the effects of environmental pH and intrinsic properties of SCCAs on antimicrobial and antibiofilm activity against *S. enterica*. We chose 21 SCCAs that represented a broad range of pK_a_ (as an indicator of acid dissociation strength, range from 1.46 to 6.60), log *K*_ow_ (as an indicator of hydrophobicity, range from −1.64 to 1.92), and molecular structures that include SCCA with additional hydroxyl, carboxyl, methyl, methylene, benzene group, and/or double bond (Table 1). Three strains of *S. enterica* subsp. *enterica* serovar Typhimurium and Enteritidis were challenged with SCCA during planktonic growth, biofilm formation, and in eradication assays.

**TABLE 1:** Activity of SCCA against planktonic growth and biofilm formation of *S. enterica^a^*

Carbon chain length	Short-chain carboxylic acids	pK_a_	Log K_ow_	Additional side groups	MIC_50_ (mM)			MBIC (mM)		
					DSM 17058	CCM 3812^*d*^	CCM 4420	DSM 17058	CCM 3812	CCM 4420
1	Formic	3.75	−0.54	N/A	7.0 ± 1.6 ^eA*b*^	1.4 ± 0.5 ^aB^	2.3 ± 0.7 ^aB*c*^	20.8 ± 6.5^dA^	3.1 ± 1.7 ^aB^	3.9 ± 1.3 ^abB^
2	Acetic	4.76	−0.17	N/A	4.6 ± 1.5 ^cdeA^	1.2 ± 0.9 ^aB^	2.0 ± 0.6 ^aB^	10.0 ± 3.4 ^bcA^	2.3 ± 0.9 ^aA^	10.7 ± 10.2 ^bA^
	Oxalic	1.46, 4.40	−0.81	―COOH	> 50	> 50	33.9 ± 16.3 ^c^	> 50	> 50	> 50
	Phenylacetic	4.31	1.41	―C_6_H_5_	2.6 ± 0.8 ^abcA^	1.2 ± 0.3 ^aB^	1.1 ± 0.2 ^aB^	6.3 ± 0.0 ^abcA^	1.6 ± 0.0 ^aA^	4.7 ± 5.3 ^abA^
3	Propionic	4.88	0.33	N/A	5.2 ± 0.8 ^deA^	1.4 ± 0.5 ^aB^	1.1 ± 0.8 ^aB^	11.5 ± 2.6 ^bcA^	2.5 ± 0.8 ^aB^	6.4 ± 8.8 ^abAB^
	Lactic	3.86	−0.72	―OH	> 50	47.7 ± 3.8 ^f^	> 50	> 50	> 50	> 50
	3-Hydroxypropionic	4.2	−0.95	―OH	14.4 ± 1.9 ^fA^	8.9 ± 2.6 ^abA^	11.1 ± 2.8 ^abA^	> 50	20.8 ± 7.2 ^b^	21.9 ± 6.3 ^c^
	3-Phenyllactic	4.02	1.18	―C_6_H_5_,	5.2 ± 1.0 ^deA^	3.5 ± 1.2 ^aB^	3.5 ± 0.4 ^aB^	10.7 ± 4.4 ^bcA^	4.9 ± 1.7 ^aB^	5.6 ± 4.7 ^abAB^
				―OH						
	Isobutyric	4.84	0.94	―CH_3_	5.8 ± 0.5 ^deA^	1.4 ± 0.6 ^aB^	3.5 ± 2.6 ^aAB^	16.7 ± 7.2 ^cdA^	2.1 ± 0.9 ^aB^	4.2 ± 1.8 ^abB^
	Malonic	2.85, 5.70	−0.81	―COOH	> 50	16.4 ± 8.9 ^bc^	28.4 ± 18.5 ^c^	> 50	> 50	> 50
4	Butyric	4.82	0.79	N/A	5.9 ± 0.4 ^deA^	1.7 ± 0.4 ^aB^	2.3 ± 0.6 ^aB^	12.5 ± 0.0 ^bcdA^	2.6 ± 0.9 ^aB^	3.5 ± 2.0 ^abB^
	Isovaleric	4.77	1.16	―CH_3_	4.9 ± 0.3 ^cdeA^	1.4 ± 0.4 ^aB^	2.2 ± 0.5 ^aB^	16.7 ± 7.2 ^cdA^	4.2 ± 1.8 ^aB^	3.9 ± 1.6 ^abB^
	Succinic	4.21, 5.64	−0.59	―COOH	> 50	24.6 ± 9.7 ^cd^	> 50	> 50	> 50	> 50
	Crotonic	4.82	0.72	N/A	0.7 ± 0.2 ^aA^	0.4 ± 0.1 ^aB^	0.2 ± 0.1 ^aC^	1.3 ± 0.4 ^aA^	1.1 ± 1.0 ^aA^	1.0 ± 1.1 ^aA^
	Tartaric	2.72, 4.34	−0.76	―COOH, ―OH	> 50	24.4 ± 8.8 ^c^	> 50	> 50	33.3 ± 14.4 ^c^	> 50
	Malic	3.51, 5.03	−1.26	―COOH, ―OH	> 50	37.0 ± 7.5 ^de^	>50	> 50	> 50	> 50
	Itaconic	3.65, 5.55	0.05	―COOH, ―CH_2_	> 50	19.0 ± 1.6 ^bc^	22.9 ± 4.8 ^bc^	> 50	> 50	> 50
5	Valeric	4.84	1.39	N/A	3.5 ± 0.8 ^bcdA^	1.3 ± 0.3 ^aB^	1.5 ± 0.3 ^aB^	12.5 ± 0.0 ^bcdA^	1.6 ± 0.0 ^aC^	3.1 ± 0.0 ^abB^
	Citric	3.13, 4.76, 6.60	−1.64	―COOH, ―OH	> 50	24.5 ± 8.6 ^cd^	22.3 ± 16.8 ^bc^	> 50	> 50	> 50
	Isocitric	3.29, 4.71, 6.40	−1.4	―COOH, ―OH	> 50	42.4 ± 13.4 ^e^	> 50	> 50	> 50	> 50
6	Caproic	4.88	1.92	N/A	2.0 ± 0.3 ^abA^	0.9 ± 0.3 ^aB^	1.3 ± 0.3 ^aB^	3.6 ± 1.3 ^abA^	1.7 ± 0.8 ^aB^	2.2 ± 0.8 ^abAB^

^*a*^ Minimum inhibitory concentration (MIC) and minimum biofilm inhibitory concentration (MBIC) were determined with twofold broth dilution assays. The highest concentration of SCCA was 50 mM, and the pH of the medium was adjusted to pH 4.5. Planktonic growth was examined at optical density at 600 nm and the minimum inhibitory concentration to reduce 50% of cell density (MIC_50_) was calculated with four-parameter logistic regression. Biofilm mass was stained with crystal violet, and the MBIC to inhibit biofilm formation (fold change < 0.1) was recorded.

^*b*^ Different superscript letters (^a–f^) indicate significant differences within the column by Tukey’s test (*P* <0.05).

^*c*^ Different superscript letters (^A–C^) indicate significant differences within the strains at pH 4.5 by Tukey’s test (*P* <0.05). Data are shown as mean ± SD (*n* ≥ 3).

^*d*^ CCM, Czech Collection of Microorganisms.

## MATERIALS AND METHODS

### Bacterial growth and culture conditions

*Salmonella enterica* subsp. *enterica* serovar Typhimurium DSM 17058, *S*. *enterica* subsp. *enterica* serovar Typhimurium CCM 3812 and *S*. *enterica* subsp. *enterica* serovar Enteritidis CCM 4420 were obtained from the Deutsche Sammlung von Mikroorganismen und Zellkulturen GmbH and Czech Collection of Microorganisms. Strains were stored as glycerol stock cultures at −80°C. To obtain working cultures, strains were streaked on Luria-Bertani (LB) (Sigma-Aldrich, USA) agar plates and activated twice in LB broth with 1% inoculum (vol/vol) at 37°C for 24 h before the experiments. The initial cell counts of *S. enterica* DSM 17058, CCM 3812, and CCM 4420 before dilution were 8.8 ± 0.0, 8.6 ± 0.1, and 8.5 ± 0.4 log CFU/mL, respectively.

### Preparation of SCCA stock solutions and SCCA characteristics

SCCA stock solutions were prepared by adding 100 mM of SCCA (listed in Table 1) to the LB broth. The final pH was adjusted to 4.5, 5.5, or 6.5 with 10N NaOH solution. After filtration (0.2 µm filters), acid stock solutions were stored at –20°C until subsequent use. pK_a_ and log *K*_ow_ (Table 1) were retrieved from the PubChem database, HMDB database, and Aurich et al. (17).

### Minimum inhibitory concentration determination with broth dilution method

A PIPETMAX automated pipetting robot (Gilson Inc., USA) was used to conduct minimum inhibitory concentration (MIC) assays. Briefly, acid stock solution (100 µL) was added to 100 µL LB broth (adjusted to pH 4.5, 5.5, or 6.5) in the second column of the 96-well microtiter plate, and a series of twofold dilutions starting from 50 mM (0.19%–1.29%) to 0.1 mM (0.0004–0.0026%) was performed. Fifty millimolar was chosen because SCCA concentrations in fermented foods are usually around or below this level; 50 mM is also within the range of 0.5%–2% SCCA (25–300 mM) that confer antimicrobial effects in most studies (6–10). Overnight cultures were centrifuged (4,000 rpm, 10 min) and resuspended in sterilized LB broth. After dilution of the washed working cultures in fresh medium (1:100), cultures were inoculated at 10% (vol/vol) into the wells to obtain a final cell concentration of ~5 log CFU/mL. The first column was used as a blank control without acid treatment and cells, while the last column was a positive control, where *S. enterica* grew without acid treatment. After incubation at 37°C for 24 h, the optical density at 600 nm (OD_600_) was recorded with an Infinite M200 Pro microplate reader (Tecan Trading AG, Switzerland). All experiments were conducted at least in three biological replicates.

### Crystal violet stain of biofilm cells and determination of minimum biofilm inhibitory concentration

After recording the OD_600_, supernatants were removed from 96-well microtiter plates to discard planktonic cells. To determine the minimum biofilm inhibitory concentration (MBIC), each well was gently rinsed with 200 µL milli-Q water, and biofilms were stained with 125 µL 0.1% crystal violet solution (in water) for 10 min (18). After removing the crystal violet solution, each well was rinsed again with 200 µL milli-Q water, and the plates were immerged in a water tray to remove excess crystal violet. The plates were pat-dried on paper towel, turned upside down, and dried overnight at room temperature. Each well was dissolved in 200 µL 96% ethanol (19) with shaking for 15 min, then the OD_595_ was recorded. Biofilm fold change was calculated as (OD_595_ of sample – OD_595_ of blank control) / (OD_595_ of negative control – OD_595_ of blank control). The MBIC was defined as the concentration that resulted in a biofilm fold change of <0.1. All experiments were conducted at least in three biological replicates.

### Minimum biofilm eradication concentration determination after a first acid treatment

To determine the minimum biofilm eradication concentration (MBEC), formic, acetic, propionic, 3-phenyllactic, crotonic, and caproic acid were selected due to major antimicrobial activity against planktonic cells. *S. enterica* was subjected to two SCCA exposures to evaluate the MBEC since previous studies suggested that a single SCCA treatment was not sufficient to reduce biofilm of *Salmonella* (20). The method was modified from Nielsen et al. (21) and Paytubi et al. (22). Briefly, twofold dilutions of SCCA (maximum 50 mM, adjusted at pH 4.5) were performed from the second to the last column in the 96-well plates by using the pipetting robot. *Salmonella* working cultures were inoculated at 10% (vol/vol). Microtiter plates were incubated at 37°C for 24 h to allow biofilm formation, where untreated cells were included for comparison. This was considered the first acid/control treatment. To test the MBEC, the supernatants containing planktonic cells were removed from each well, and the biofilm cells in the microtiter plates were subjected to a second acid treatment with twofold dilution series of SCCA (maximum 50 mM, adjusted at pH 4.5) from the first to the last row. Both untreated and SCCA-treated biofilms were tested for MBEC. The OD_600_ was measured before and after cultivating plates at 37°C for 24 h. The lowest concentration of SCCA that led to: OD_600_ after incubation – OD_600_ before incubation <0.1 was defined as the MBEC. All experiments were conducted in at least three biological replicates.

### Statistical analysis

The OD_600_ values generated from MIC assays were fitted into a four-parameter logistic regression (sigmoidal curve) to calculate the minimum inhibitory concentration to reduce 50% of cell density (MIC_50_) (23) using GraphPad Prism version 8 (GraphPad Software Inc., USA) (Table 1). One-way analysis of variance and Tukey’s test (multiple-group comparison), and Student’s *t*-test (two-group comparison) were computed with R software to compare the MIC_50_, MBIC, and MBEC values. Correlation analysis was conducted with R packages Hmisc and ggcorplot. A Spearman’s correlation test was chosen because the data were not normally distributed as shown in the Shapiro-Wilk normality test (Table S3). Correlation coefficients of 0.20–0.39, 0.40–0.59, and 0.60–0.79 indicated weak, moderate, and strong relationships, respectively. All test results with *P* <0.05 were considered as significantly different, while 0.05< *P* <0.1 was considered a trend.

## RESULTS

### Impact of SCCAs on planktonic growth of *S. enterica*

In this study, we investigated the antimicrobial activity of SCCA against *S. enterica*, which remains a major threat in causing foodborne diseases. *S. enterica* was cultivated in the 96-well plates at 37°C for 24 h in the presence of SCCA using a twofold broth dilution assay. The pH of the medium was adjusted to pH 4.5, 5.5, and 6.5 since the pH value of the (fermented) food product ranges from acidic to acidic/neutral; fermented foods of longer shelf-life usually have pH values of ≥4.2.

**Fig 1:**
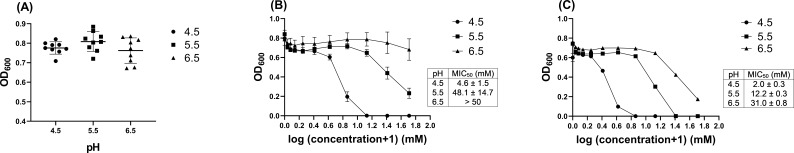
Impact of pH levels and SCCAs on planktonic growth of *S. enterica* DSM 17058. *S. enterica* DSM 17058 was grown at 37°C for 24 h to evaluate the effect of (**A**) pH, (**B**) acetic and (**C**) caproic acid on optical density. SCCAs were diluted twofold (0.1–50.0 mM), and the medium pH was adjusted to pH 4.5, 5.5, and 6.5.

The growth of *S. enterica* DSM 17058 was similar at all three pH values with an average OD_600_ of 0.78 ± 0.03, 0.81 ± 0.05, and 0.76 ± 0.07 at pH 4.5, 5.5, 6.5, respectively, after 24 h of incubation (Fig. 1A). *S. enterica* DSM 17058 was inhibited by SCCA in a pH-dependent manner with 57% (12 of 21), 33% (7 of 21), and 10% (2 of 21) of the tested SCCA with MIC_50_ lower than 50 mM at pH 4.5, 5.5, and 6.5, respectively (Table S1). Addition of acetic acid reduced the cell density to 50% or less of *S. enterica* DSM 17058 at pH 4.5 (MIC_50_ = 4.6 mM) and 5.5 (MIC_50_ = 48.1 mM) but not at pH 6.5 (Fig. 1B). A similar pattern was observed for phenylacetic, 3-phenyllactic, propionic, and valeric acids at pH 4.5 (MIC_50_ = 2.6–5.2 mM) and 5.5 (MIC_50_ = 18.6–49.9 mM). Formic, butyric, 3-hydroxypropionic, isobutyric, and isovaleric acids only conferred detectable inhibition at pH 4.5 (MIC_50_ = 4.9–14.4 mM). Crotonic and caproic acids were the only SCCAs that reduced the growth of *S. enterica* DSM 17058 at all three tested pH values. The MIC_50_ of caproic acid was 15-fold higher at pH 6.5 than 4.5 (31 versus 2.0 mM ) (Fig. 1C).

Sensitivity of two more strains of *S. enterica*, CCM 3812 and CCM 4420, was tested at pH 4.5 as we observed the highest antimicrobial activity at acidic pH for *S. enterica* DSM 17058. The growth rates of three *S*. *enterica* strains (Fig. S1) were not significantly different (*P* >0.05, *k* = 0.77–1.21 h^−1^). Planktonic cell growth of *S. enterica* CCM 3812 and CCM 4420 was inhibited by 95% (20 of 21) and 76% (16 of 21) of SCCA with MIC_50_ values lower than 50 mM, respectively (Table 1), which indicates higher sensitivity compared to DSM 17058 (57%, 12 of 21). *S. enterica* CCM 3812 and CCM 4420 were more vulnerable to acid stress since their MIC_50_ values were 1.8 ± 1.6 times lower than DSM 17058. In contrast to *S. enterica* DSM 17058, SCCA with two or more carboxyl groups such as succinic and citric acids reduced growth of *S. enterica* CCM3812 and CCM 4420.

In general, SCCAs with pK_a_ values lower than 4.0 including lactic acid, and SCCAs with two or more carboxylic groups (e.g., oxalic, malic, and isocitric acids) had less or no effect to inhibit three strains of *S. enterica* (MIC_50_ >30 mM). The only exception was formic acid (pK_a_ = 3.75) with a MIC_50_ of 1.4–7.0 mM.

### Impact of SCCAs on biofilm formation of *S. enterica*

Next, we investigated the antibiofilm activity of SCCA against *S. enterica*, which is frequently found as biofilm attachment on food surfaces and equipment of food processing plants (24). Biofilms were grown in 96-well microtiter plates for 24 h in the presence of SCCA (maximal concentration was 50 mM).

**Fig 2:**
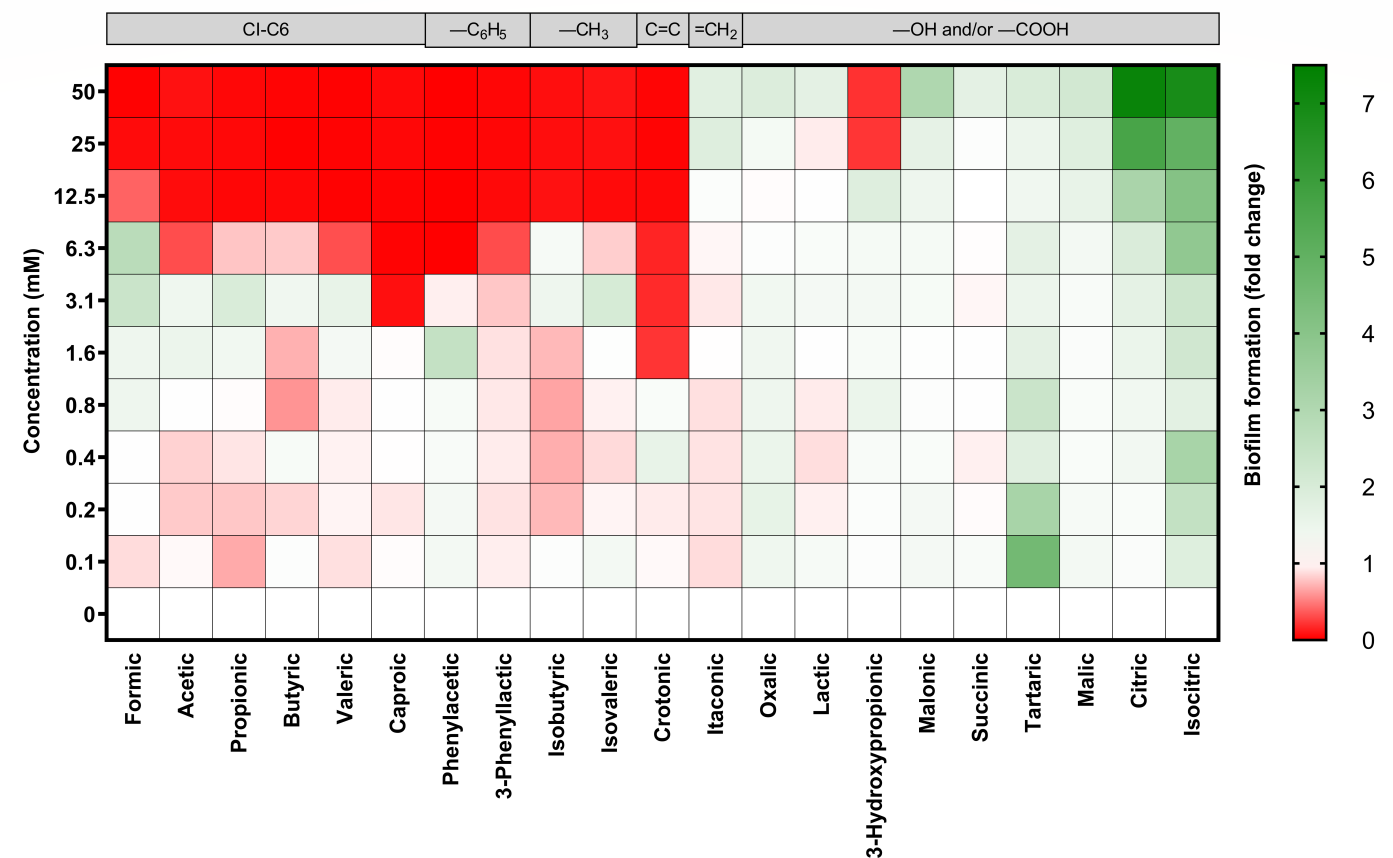
Impact of SCCA on biofilm formation of *S. enterica* DSM 17058. SCCAs were diluted twofold for final concentrations of 0.1–50.0 mM, and the medium pH was adjusted to pH 4.5. The biofilm mass was determined with crystal violet stain. Background colors filled with red and green represent biofilm inhibition and increment, respectively. Gray boxes indicate the structural properties of SCCA.

Surprisingly, we observed the stimulation of biofilm formation for *S. enterica* DSM 17058 at pH 4.5 at certain SCCA concentrations close to MIC_50_ (Fig. 2). Formic, acetic, and propionic acids increased biofilm formation two- to threefold compared to untreated controls at 6.3 mM (MIC_50_ = 7.0 mM), 1.6 mM (MIC_50_ = 4.6 mM), and 3.1 mM (MIC_50_ = 5.2 mM), respectively. Inhibition was only observed at 12.5 mM or higher. Citric and isocitric acids did not inhibit planktonic growth of DSM 17058 but enhanced biofilm formation especially at the highest 50 mM.

Due to the stimulation of biofilm formation at specific conditions, we could not calculate the MBIC_50_ value using sigmoidal curve fitting and recorded the MBIC as the lowest concentration that resulted in biofilm fold change of <0.1 after incubation.

At pH 4.5, biofilm formation of the three *Salmonella* strains was inhibited by 52%–62% (11 of 21 to 13 of 21) of the tested SCCAs (MBIC <50 mM) (Table 1). SCCA with one to six carbons in the backbone and SCCA with additional benzene (―C_6_H_5_, e.g., phenylacetic and 3-phenyllactic acids) and methyl (―CH_3_, e.g., isobutyric and isovaleric acids) groups or a double bond (e.g., crotonic acid) inhibited biofilm formation of *S. enterica* DSM 17058 at a MBIC of 1.6–12.5 mM (Table 1). Crotonic acid was the most effective SCCA to avoid *S. enterica* biofilm at pH 4.5 (MBIC = 1.3 mM), followed by caproic (MBIC = 3.6 mM) and phenylacetic (MBIC = 6.3 mM) acids (*P* <0.05). These SCCAs did not allow biofilm formation at any of the concentrations that were tested. In contrast to DSM 17058, CCM 3812 and CCM 4420 were inhibited by 3-hydroxypropionic acid. Biofilms of CCM strains were more susceptible to SCCA as indicated by MBIC values that were 3.2 ± 2.3 times lower than of DSM 17058.

SCCA with additional an carboxyl and/or hydroxyl group [e.g., lactic, oxalic, and malonic acids (MBIC ≥50 mM)] had less or no effect on *Salmonella* biofilm formation (Table 1). Other SCCAs with additional carboxyl groups, such as succinic, malic, and citric acids, did not interfere with biofilm formation of *S. enterica*. Although the absolute MIC_50_ and MBIC differed between strains, the overall sensitivity of strains toward the structurally different SCCA was similar. Compared to the MIC_50_ of planktonically growing cells at pH 4.5, the MBIC of *S. enterica* was 1.2–5.8 times higher.

At pH 5.5 and 6.5, biofilm inhibition was tested only for *S. enterica* DSM 17058. Caproic, crotonic, and phenylacetic acids inhibited biofilm formation at concentrations between 25 and 50 mM (Fig. S2).

### The potential of SCCA to eradicate *S. enterica* biofilm

In this study, we tested the ability of formic, acetic, propionic, 3-phenyllactic, crotonic, and caproic acids, which were among the most active SCCAs (Table 1) to eradicate biofilms by two consecutive acid treatments. *S. enterica* DSM 17058, CCM 3812, and CCM 4420 biofilms were grown in 96-well plates for 24 h with SCCA concentrations ranging from 0.78 to 50.0 mM (first acid treatment). After 24 h of incubation, the supernatants were removed, and the grown biofilm cells were subjected to a second SCCA treatment (second acid treatment, 0.4- to 50.0-mM SCCA) to test for biofilm eradication. The MBEC was identified as the lowest concentration leading to an OD_600_ of <0.1 after two SCCA exposures.

**Fig 3:**
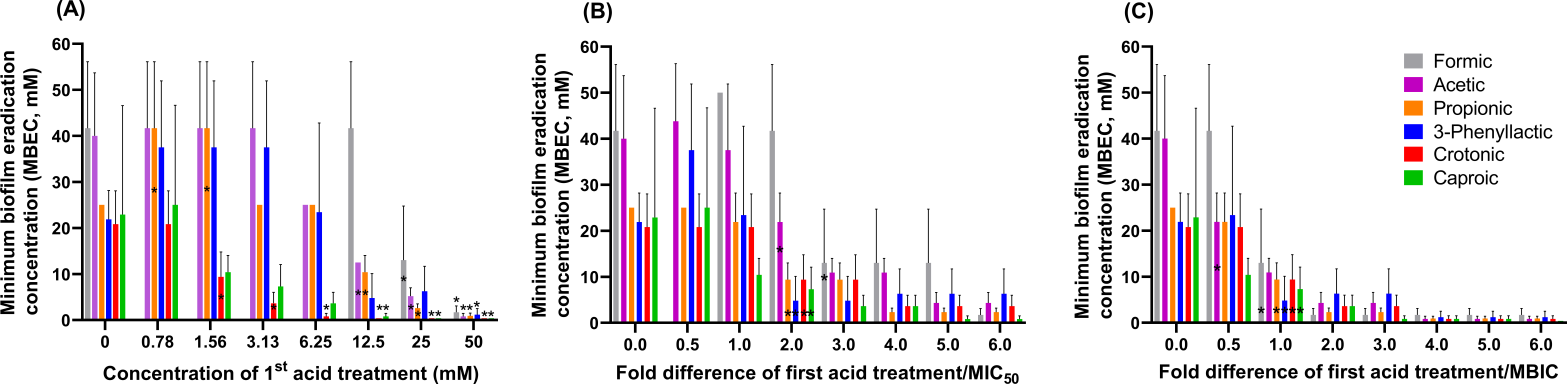
Ability of SCCA to eradicate biofilm cells of *S. enterica* DSM 17058 with two consecutive SCCA exposures. Biofilms were grown with SCCA (first acid treatment), then the biofilm cells were subjected to second acid treatment to test the minimum biofilm eradication concentration (MBEC). Relationships of MBEC and first acid treatment related to (**A**) SCCA concentrations, (**B**) MIC_50_, and (**C**) MBIC are presented. MBEC values of formic acid were higher than 50 mM when added in the first treatment at levels of 0.78–6.25 mM, and were not included in Fig. 1A. *Significant difference (*P* < 0.05) when compared to the corresponding untreated control at zero concentration with Student’s *t*-test.

When there was no SCCA present in the first treatment (untreated control), the MBEC was ~20 mM for propionic, 3-phenyllactic, crotonic, and caproic acids for all three strains of *S. enterica*, which was significantly lower than that for formic and acetic acid (~40 mM, *P* <0.05) (Table S2). Since MBEC values were comparable for all three strains (Table S2), we focused on the more SCCA-resistant strain, DSM 17058, when investigating the impact of previous SCCA exposure (first acid treatment) . While the MBECs of crotonic and caproic acids against *S. enterica* DSM 17058 biofilm were similar to the untreated control when the first acid treatment was 0.78 mM, the MBECs were significantly lower when acid concentrations were 1.56 mM and higher during the first treatment (Fig. 3A). The presence of low concentrations of propionic acid during the first treatment (0.78 and 1.56 mM) led to a higher MBEC (~40 mM) compared to the untreated control (~20 mM) (*P* <0.05). The MBEC of propionic acid significantly was significantly lower compared to untreated controls only when added at concentrations of 12.5 and 25 mM in the first treatment. A similar trend (*P* = 0.08) was observed for 3-phenyllactic acid. For acetate and formate, a first acid treatment of 12.5 and 25 mM led to a significant reduction of the MBEC compared to untreated controls.

When relating the MIC_50_ values to the concentration of the first acid treatment (Fig. 3B), we observed that caproic acid showed an MBEC of ~10 mM when *S. enterica* DSM 17058 was exposed to levels similar to the MIC_50._ Formic and acetic acids required concentrations that were at least 3× MIC_50_ to reduce MBEC below 20 mM, and 6× MIC_50_ for an MBEC of ≤10 mM, respectively. Similar observations were made if the MBIC was related to the MBEC (Fig. 3C). The tested SCCA significantly reduced the MBCE compared to the untreated control *(P* <0.05) when present at levels similar to the MBIC. The MBEC was even lower (~5 mM) if SCCAs were added at 2× to 3× MBIC. Biofilm cells were eradicated when SCCA were present at 4× MBIC.

These results point out that levels of SCCA in the first acid treatment were crucial to ensure biofilm eradication by the second acid treatment. Caproic and crotonic acids were the strongest biofilm eradicators, while propionic acid could lead to higher MBEC values if added at too low concentration in the first treatment.

### Associations between environmental conditions, SCCA intrinsic parameters, MIC_50_, and MBIC

To explore the relationship of intrinsic properties of SCCA and environmental pH with inhibition of planktonic growth (represented by the MIC_50_) and biofilm formation (represented by the MBIC) of *S. enterica*, Spearman’s correlation test was conducted (Fig. S3). Relevant factors were the presence of side groups (e.g., hydroxyl, carboxyl, methyl, methylene, and benzene groups), the presence of a double bond, the log *K*_ow_ as an indicator of hydrophobicity, and the pK_a_.

MIC_50_ and MBIC had a strong positive correlation (*r* = 0.93). MIC_50_ or MBIC correlated strongly with the surrounding pH (*r* = 0.64, 0.60) and moderately with the absence of additional carboxyl group(s) (*r* = 0.53, 0.52). There was a moderate correlation of MIC_50_ or MBIC with the pK_a_ (*r* = –0.54, –0.51) and log *K*_ow_ (*r* = –0.53, –0.53). The log *K*_ow_ was related to the presence of SCCA side groups, especially to additional carboxyl (―COOH, *r* = –0.72), hydroxyl (―OH, *r* = –0.49), and benzene (―C_6_H_5_, *r* = 0.43) groups. SCCAs with additional carboxyl and/or hydroxyl group (e.g., lactic, succinic, tartaric, malic, and citric acids) had negative log *K*_ow_ values and showed higher MIC_50_ values than SCCA with positive log *K*_ow_.

**Fig 4:**
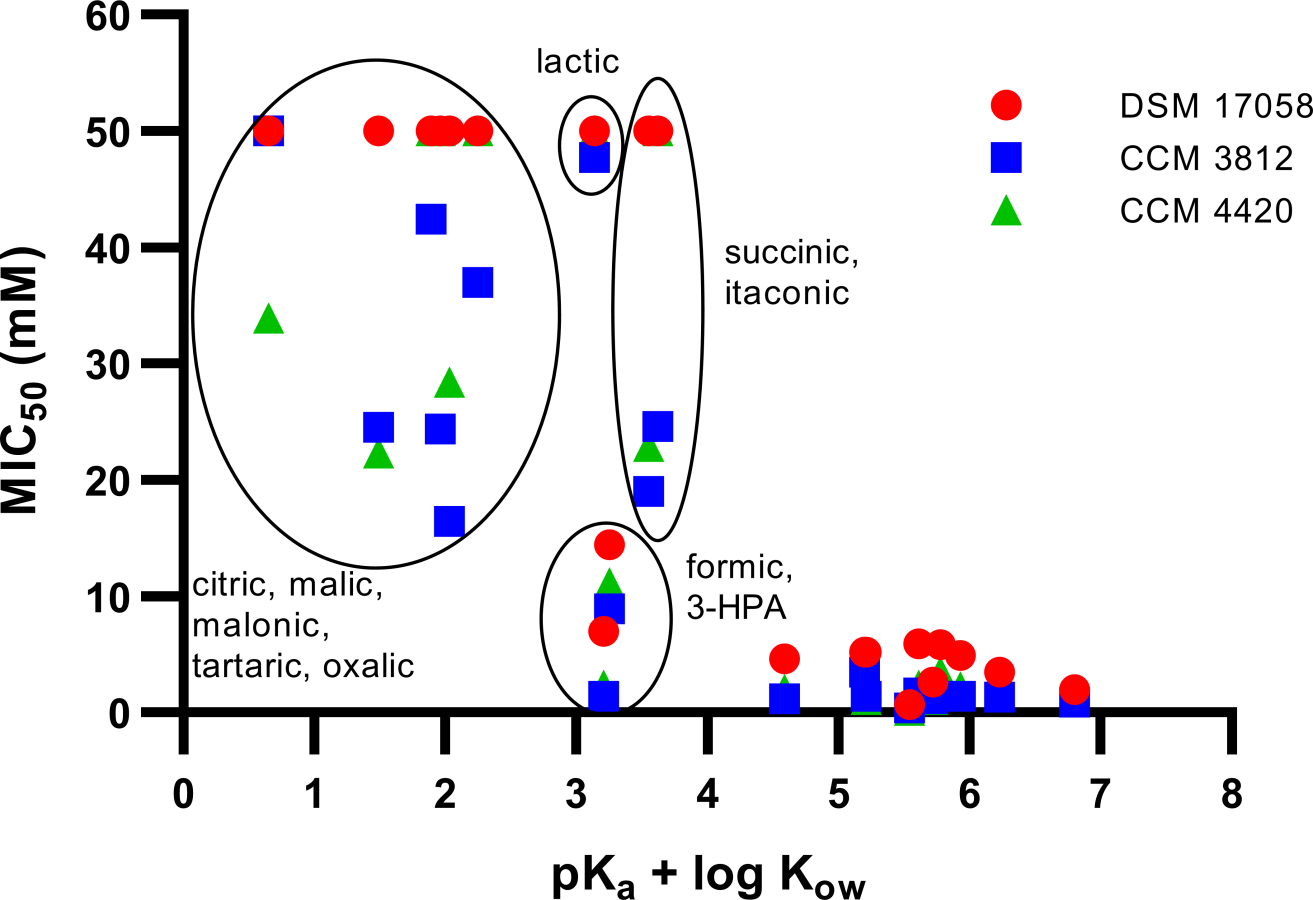
Combined effect of pK_a_ and log *K*_*o*w_ towards SCCA antimicrobial activity. The sum of pK_a_ and log *K*_ow_ was calculated and depicted against the MIC_50_ of SCCAs against strains DSM 17058, CCM 3812 and CCM 4420. SCCAs with MIC_50_ of >50 mM were plotted as 50 mM.

When we calculated the sum of pK_a_ and log *K*_ow_, any SCCA that led to pK_a_ + log *K*_ow_ >4 demonstrated antimicrobial activity against all three strains of *Salmonella* (MIC_50_ of <6 mM), suggesting a parameter predicting the outcome of inhibition (Fig. 4). For SCCA with 3< pK_a_ + log *K*_ow_ <4 and pK_a_ + log *K*_ow_ <3, the antimicrobial activity was much more variable. The MIC_50_ of formic (pK_a_ + log *K*_ow_ = 3.21) and 3-hydroxypropionic acids (3.25) ranged from 2.3 to 14.4 mM, while the MIC_50_ of lactic acid (3.14) was between 47.7 and >50 mM. For citric, malic, malonic, tartaric, and oxalic acids with low pK_a_ + log *K*_ow_ of 0.65–2.55, MIC_50_ ranged from 16.4 to >50 mM, depending on the strain that was tested.

## DISCUSSION

### Applicability of SCCA in food biopreservation

This study indicated that SCCAs differ in antimicrobial and antibiofilm activity depending on compound structure and environmental conditions. In most food fermentations, lactic, acetic, and propionic acids are the major SCCAs that are formed, while phenyllactic acid occurs at low concentration and caproic acid was reported for only few food types (25).

*Lactobacillaceae* have a long history as starter cultures for food fermentation, and lactic acid is the major fermentation metabolite that is responsible for reducing the environmental matrix pH of final fermented food products to 4.0–4.5. We observed here that the direct antimicrobial effect of lactic acid against cells of *S. enterica* was negligible if the pH was controlled, suggesting that the main antimicrobial function of lactic acid relates to the ability to lower food matrix pH and not to direct compound-microbe interactions. These observations might explain why high concentrations of lactic acid (134 mM for chicken meats) (12) and combined treatments such as lactic acid with steam gas or UV light (14, 15) were needed to reduce *Salmonella* counts in previous studies. Our results were similar to those of Liang et al. (23), who found that lactic acid (up to 640 mM) compared to propionic acid could not inhibit yeasts and fungi (23).

In contrast, propionic and caproic acids showed stronger antimicrobial activity than lactic acid at controlled pH likely due to direct interactions affected by pK_a_ and the hydrophobic compound structure. Formation of propionic acid is exploited in food biocontrol with co-cultures of *Propionibacterium jensenii* and *Lacticaseibacillus paracasei* that form propionic and acetic acids from lactic acid cross-feeding (26, 27). This consortium also produced phenyllactic acid at levels that were too low to be responsible for inhibition of yeast (1 mM). Similarly, low levels were formed in sourdough (0–0.3 mM) and kimchi (0.06 mM).

Very few studies reported the formation of the strong antimicrobial caproic acid in food and beverages; for example, caproic acid has been detected in some Chinese liquor fermented with glucose and lactose (28, 29). Caproic acid is characterized by a distinctive goat-like odor. In our study, the MIC_50_ for caproic acid was around 1–2 mM (0.0125%–0.015%), which is 25–30 times higher than the suggested sensory threshold at 0.0005%. Nevertheless, a sensory threshold might be altered depending on the food matrix, and is usually higher in a food product than in water (30, 31). Running and Mattes (32) reported an oral threshold of 1.45 mM caproic acid when tested in gums (32). Similar levels are desired in goat cheeses to provide the characteristic sensory profiles (33).

### Certain SCCAs act as antimicrobials at near-neutral pH

Our results show that SCCAs inhibited both planktonic and biofilm cells more effectively at lower environmental pH that is commonly observed in fermented food products. The amount of undissociated acid increases with decreasing pH and higher pK_a_ according to the Henderson-Hasselbalch equation. The weak acid theory suggests that undissociated acids are lipid permeable and could permeate through the cell barrier to induce cytoplasmic acidification and turgor pressure (34), which contributes to the stronger antimicrobial effect or lower MIC_50_ at pH 4.5 or pK_a_ >4.0 observed in our results.

Although low pH enhanced the antimicrobial activity of SCCA, crotonic and caproic acids inhibited *S. enterica* growth even at pH 6.5. Crotonic and caproic acids have a higher proportion of dissociated acids at pH 6.5 (~98%) than at pH 5.5 (~80%) and 4.5 (~30%), which implies that the antimicrobial activity of SCCA can be attributed to dissociated or undissociated acids, or a combination of both, particularly at near a neutral pH. In agreement, Pernin et al. (35) proposed that extracellular dissociation of the phenolic acids, p-coumaric acid, and ferulic acid significantly contributed to the inhibition against *Listeria monocytogenes* at a near-neutral pH of 7.2 possibly due to damage of cell membrane (35).

It was suggested that carboxylic acids act as chelating agents of divalent cations such as Mg^2+^ and Ca^2+^ to destabilize the outer membrane of Gram-negative *Salmonella* by disintegrating the lipopolysaccharides (36). Burel et al. (37) proposed that the tribasic form of citric acid (CA^3−^) was responsible for the toxicity toward Gram-negative bacteria at pH 6.5 because of metal ion chelation and the decrease of bacterial surface charge compared to pH 4.5, which might render the cells even more sensitive against chelants (37).

Taken together, these results suggest that low pH (~4.5) and high pK_a_ increased the antimicrobial activity mainly due to the presence of undissociated acids that penetrated through the cells, while at near-neutral pH (~6.5 to 7.0), the presence of dissociated crotonic and caproic acids might additionally affect cell membrane integrity.

### Hydrophobicity together with the pK_a_ define the effectiveness of individual SCCA as antimicrobial and antibiofilm agents

Crotonic, caproic, propionic, valeric, and phenylacetic acids outperformed the other SCCAs as antimicrobials and antibiofilm agents. These SCCAs are structurally different but have in common a positive log *K*_ow_ with values ranging from 0.33 to 1.92. Caproic acid has the longest carbon chain (C6) among the tested SCCAs followed by valeric (C5) and propionic (C3) acids, while phenylacetic and crotonic acids possess a benzene ring and a double bond, respectively. Hydrophobic SCCAs have better diffusion rates through the phospholipid bilayer of microbial cells mainly when undissociated. The membrane permeability of caproic acid (C6, log *K*_ow_ = 1.92) was around 50 times higher than that of acetic acid (C2, log *K*_ow_ = – 0.17) (38). Stratford et al. (39) showed that MIC values of SCCA toward *Aspergillus niger* N402 were higher with a growing carbon number of C2–C6. At the same time the log *K*_ow_ increased from –0.17 to 1.92 (39), suggesting that the log *K*_ow_ is an important factor contributing to and determining the antimicrobial activity of SCCA.

In this study, the sum of pK_a_ + log *K*_ow_ >4.0 was a reliably predictor of antimicrobial activity against different strains of *Salmonella* despite small differences in sensitivity. For SCCAs with 3< pK_a_ + log *K*_ow_ <4, antimicrobial activity ranged from no inhibition at test conditions (lactic acid) to inhibitory potential (hydroxypropionic and formic acid), indicating that pK_a_ and log *K*_ow_ together were not the sole determinant for antimicrobial activity of SCCA. Formic acid likely does not permeate across the *Salmonella* membrane but instead is transferred into the cells by the transporter FocA when the pH is lower than 6.5 (40, 41). Lactic and 3-hydroxypropionic acids are structural isomers that only differ in the position of the hydroxyl group. A stronger antimicrobial activity of 3-hydroxypropionic acid compared to lactic acid has been observed (23).

While general responses to SCCA exposure were similar, we observed that *S. enterica* CCM 3812 and CCM 4420 were more susceptible to SCCA than DSM 17058. Differences in susceptibility of *Salmonella* strains toward SCCA could result from varying acid tolerance response (ATR) (42). ATR includes the secretion of enzymes (CadA and AdiA) for amino acid conversion that involves consumptions of proton to maintain intracellular pH, the expression of acid shock proteins for cell repairment, and/or the modification of membrane composition to maintain cell fluidity. Wang et al. (43) found that the acid resistance profiles based on small non-coding RNA (sncRNA) differed between *Salmonella* strains (43), while some sncRNAs were related to the regulation of the essential acid shock protein, RpoS.

### The presence of certain SCCAs can enhance biofilm formation

When evaluating the antibiofilm character of SCCA, we noticed that concentrations of certain SCCA close to the MIC_50_ values for planktonic cells enhanced the biofilm formation of *S. enterica* DSM 17058. *Salmonella* tends to promote biofilm formation as a protection mechanism, for example, when exposed to sublethal stresses. In the present study, formic, acetic, and propionic acids did increase biofilms. These SCCAs are common metabolites in food fermentations, and the ability to promote *S. enterica* biofilm should draw attention to the manufacturing sites of fermentation industry.

In the presence of citric acid, *S. enterica* DSM 17058 biofilm was up to sevenfold higher likely due to the catabolism of citrate in the tricarboxylic acid cycle relating to energy generation. *S. enterica* DSM 17058 (also named *S*. Typhimurium LT2) is capable of utilizing citrate under aerobic conditions (44, 45).

Together, our suggests that stimulation of biofilm can occur with sublethal stress conditions (e.g., close to MIC_50_), and specific SCCAs that can be metabolized (e.g., citric acid) by *Salmonella*. SCCAs including formic, acetic, and propionic acids should be implemented at levels that are at least two times the MIC_50_ to avoid and eradicate biofilms of three *Salmonella* strains.

### Two-step SCCA treatment strategies to eradicate *S. enterica* biofilm

Food-related biofilm formation is frequently addressed by preventing the biofilm growth and/or eliminating mature biofilm through ultrasound, ultraviolet, and chemical disinfectants (46). Here, we tested the impact of two consecutive SCCA treatments as a novel approach to eradicate *Salmonella* biofilm. The MBEC values of the potent SCCA to eliminate *S. enterica* biofilm were much lower (MBEC ≤20 mM, Fig. 3) if the cells were exposed to SCCA levels that represented two- to threefold of MIC_50_ or one- to twofold of the MBIC.

At the same time, MBEC values were higher if *S. enterica* was exposed to low concentrations of propionic or phenyllactic acids compared to untreated controls. This phenomenon could be attributed to bacterial pre-adaptation processes. If a bacteria cell is exposed temporarily to lethal or sublethal stress conditions, such pre-adaptation might enhance its survival at future stress exposure (47, 48). For example, the survival of *S. enterica* at 70°C was higher after treatment at 45°C for 3 min (49). In the current study, cellular pre-adaptation of *S. enterica* was not observed when cells were first treated with acetic, crotonic, and caproic acids, suggesting a compound and/or concentration-dependent effect.

Based on our results, this study suggests a two-step acid treatment to tackle *S. enterica* biofilm formation. Such a procedure should be generally feasible, yet concentrations of SCCA need to be considered, depending on the SCCA that is intended for use. To overcome any possible pre-adaptation effect, SCCAs have to be added at levels around two to three times of MIC_50_ concentrations at acidic pH 4.5.

## SUPPLEMENTAL MATERIAL

Supplemental material spectrum.01658-24-s0001.docx Fig. S1 to S3; Tables S1 to S3.

ASM does not own the copyrights to Supplemental Material that may be linked to, or accessed through, an article. The authors have granted ASM a non-exclusive, world-wide license to publish the Supplemental Material files. Please contact the corresponding author directly for reuse.
