# Supplementary material for: Environmental pH and compound structure affect the activity of short-chain carboxylic acids against planktonic growth, biofilm formation, and eradication of the food pathogen Salmonella enterica
Source: Microbiol Spectr. 2024 Sep 16;12(11):e01658-24. doi: 10.1128/spectrum.01658-24 (PMC11537019; doi:10.1128/spectrum.01658-24)
Supplement: Supplemental material — Fig. S1 to S3; Tables S1 to S3. [file spectrum.01658-24-s0001.docx]

**Supplementary data**

**Environmental pH and compound structure affect the activity of short-chain carboxylic acids against planktonic growth, biofilm formation and eradication of the food pathogen *Salmonella enterica***

**Ker-Sin Ng^1^, Maria Florencia Bambace^1^, Emilie Balleby Andersen^1^, Rikke Louise Meyer^2,3^, Clarissa Schwab^1,#^**

^1^ Department of Biological and Chemical Engineering, Aarhus University, Gustav Wieds Vej 10, 8000, Aarhus C, Denmark.

^2^ Interdisciplinary Nanoscience Center, Aarhus University, Gustav Wieds Vej 14, 8000, Aarhus C, Denmark.

^3^ Department of Biology, Aarhus University, Ny Munkegade 116, 8000, Aarhus C, Denmark.

^#^Address correspondence to Clarissa Schwab: [schwab@bce.au.dk](mailto:schwab@bce.au.dk)

**SUPPLEMENTARY FIGURE LEGENDS**

**FIG S1** The growth kinetics of *S. enterica* in Luria Bertani (LB) broth at 37°C. The optical density was recorded at 600 nm for 24 h. The growth rate, k, was calculated by fitting in the Gompertz growth model.

**FIG S2** Impact of SCCA on biofilm formation of *S. enterica* DSM 17058. The strain was treated with two-fold dilutions (0.1 – 50 mM) of SCCA at adjusted (**A**) pH 5.5 and (**B**) 6.5. The biofilm mass was determined with crystal violet stain. Background colours filled with red and green represent biofilm inhibition and increment respectively. Grey boxes indicate the structural properties of SCCA.

**FIG S3** Spearman’s correlation test of environmental and intrinsic properties of antimicrobial SCCA. Environmental pH, pK_a_, log K_ow_, carbon length in the backbone, and different side groups (hydroxyl, carboxyl, methyl, methylene, benzene group and/or double bond) were correlated with SCCA concentrations reducing the planktonic growth (MIC_50_) and biofilm (MBIC) formation of *S. enterica* using Spearman correlation. Correlation coefficients of 0.20 – 0.39, 0.40 – 0.59 and 0.60 – 0.79 indicate weak, moderate, and strong relationships respectively. A *P* < 0.05 was considered statistically different.

**FIGURE S1**


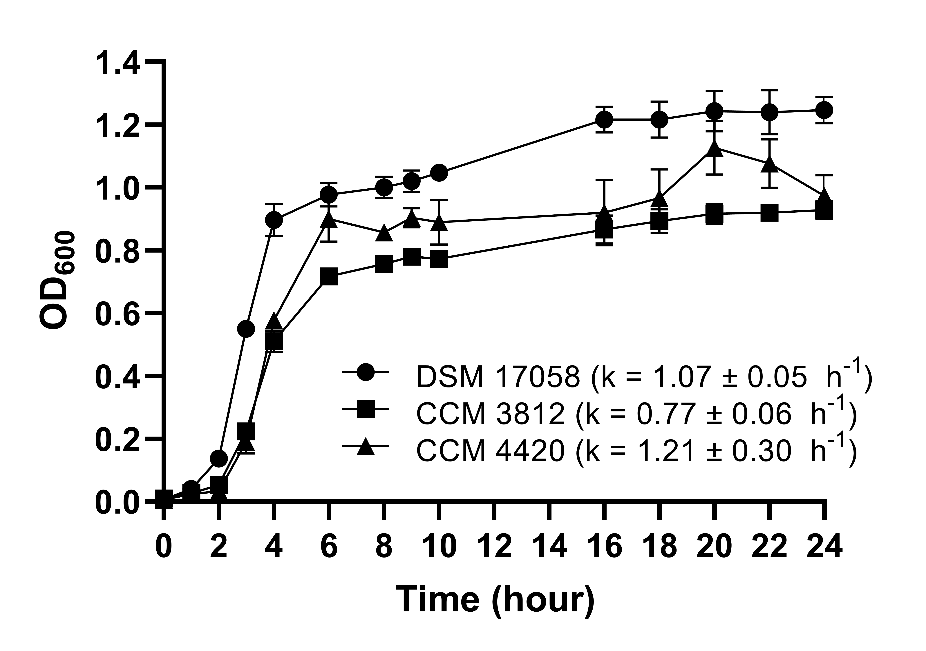


**FIGURE S2**

**FIGURE S3**


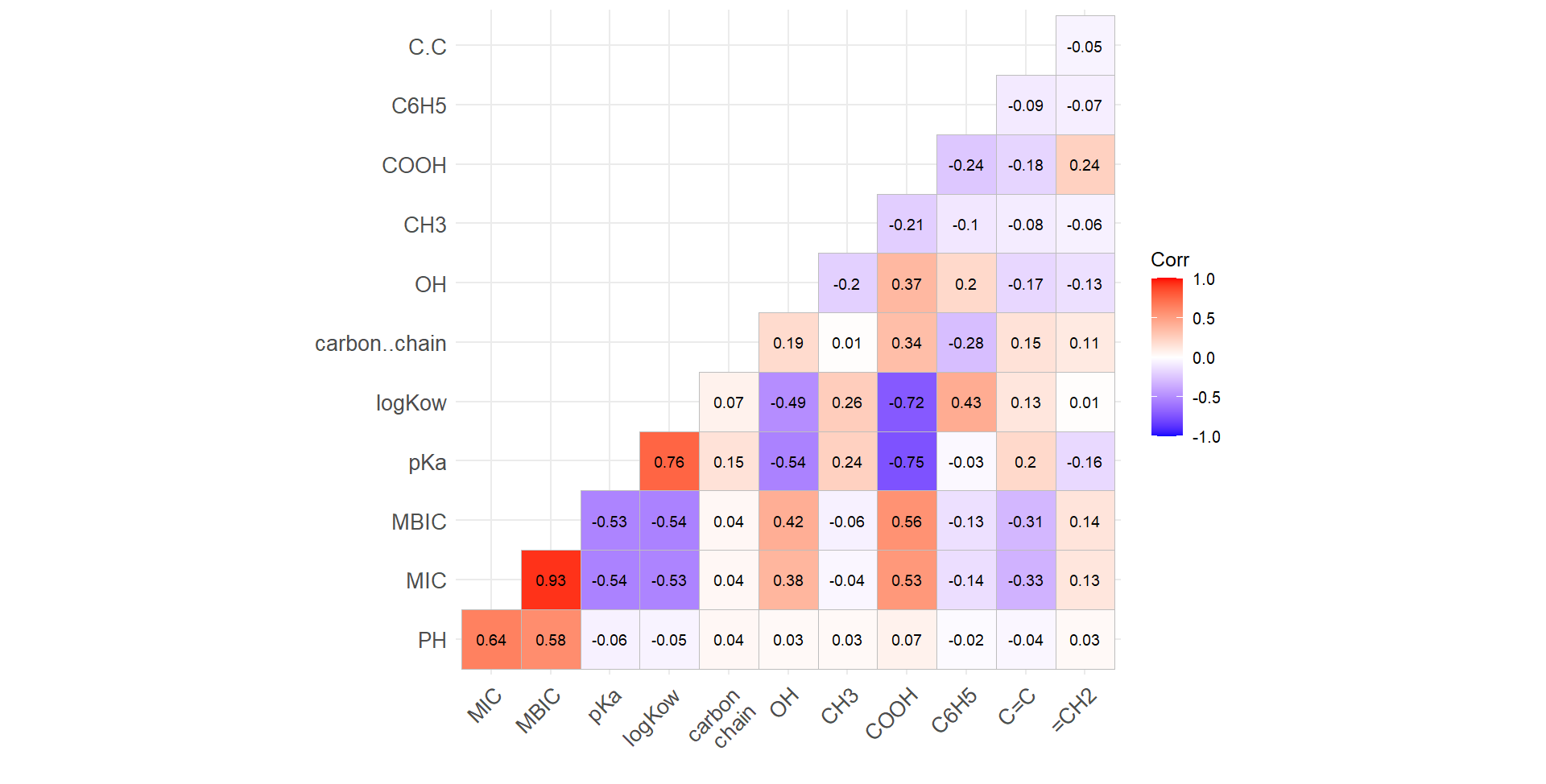


**TABLE S1** Activity of SCCA against planktonic growth and biofilm formation of *S. enterica* DSM 17058*.* Minimum inhibitory concentration (MIC) and minimum biofilm inhibitory concentration (MBIC) were determined with two-fold broth dilution assays. The highest concentration of SCCA was 50 mM, and the pH of the medium was adjusted to pH 4.5, 5.5 and 6.5. Planktonic growth was examined at OD_600_ and the MIC to reduce 50% of cell density (MIC_50_) was calculated with four-parameters logistic regression. Biofilm mass was stained with crystal violet and the MBIC to inhibit biofilm formation (fold change < 0.1) was recorded.

| **Carbon chain length** | **Short-chain carboxylic acids** | **pK_a_** | **Log Kow** | **Additional side groups** | **MIC_50_ (mM)** | | | **MBIC (mM)** | | |
| --- | --- | --- | --- | --- | --- | --- | --- | --- | --- | --- |
|  |  |  |  |  | **pH 4.5** | **pH 5.5** | **pH 6.5** | **pH 4.5** | **pH 5.5** | **pH 6.5** |
| 1 | Formic | 3.75 | -0.54 | N/A | 7.0 ± 1.6 ^e^ | > 50 | > 50 | 20.8 ± 6.5^d^ | > 50 | > 50 |
| 2 | Acetic | 4.76 | -0.17 | N/A | 4.6 ± 1.5 ^cde^ | 48.1 ± 14.7 ^cd^ | > 50 | 10.0 ± 3.4 ^bc^ | > 50 | > 50 |
|  | Oxalic | 1.46, 4.40 | -0.81 | ―COOH | > 50 | > 50 | > 50 | > 50 | > 50 | > 50 |
|  | Phenylacetic | 4.31 | 1.41 | ―C_6_H_5_ | 2.6 ± 0.8 ^abc^ | 18.6 ± 3.0 ^ab^ | > 50 | 6.3 ± 0.0 ^abc^ | 50 ± 0.0 ^b^ | > 50 |
| 3 | Propionic | 4.88 | 0.33 | N/A | 5.2 ± 0.8 ^de^ | 35.0 ± 5.4 ^bcd^ | > 50 | 11.5 ± 2.6 ^bc^ | > 50 | > 50 |
|  | Lactic | 3.86 | -0.72 | ―OH | > 50 | > 50 | > 50 | > 50 | > 50 | > 50 |
|  | 3-Hydroxypropionic | 4.20 | -0.95 | ―OH | 14.4 ± 1.9 ^f^ | > 50 | > 50 | > 50 | > 50 | > 50 |
|  | 3-Phenyllactic | 4.02 | 1.18 | ―C_6_H_5_, ―OH | 5.2 ± 1.0 ^de^ | 49.9 ± 2.0 ^d^ | > 50 | 10.7 ± 4.4 ^bc^ | > 50 | > 50 |
|  | Isobutyric | 4.84 | 0.94 | ―CH_3_ | 5.8 ± 0.5 ^de^ | > 50 | > 50 | 16.7 ± 7.2 ^cd^ | > 50 | > 50 |
|  | Malonic | 2.85, 5.70 | -0.81 | ―COOH | > 50 | > 50 | > 50 | > 50 | > 50 | > 50 |
| 4 | Butyric | 4.82 | 0.79 | N/A | 5.9 ± 0.4 ^de^ | > 50 | > 50 | 12.5 ± 0.0 ^bcd^ | > 50 | > 50 |
|  | Isovaleric | 4.77 | 1.16 | ―CH_3_ | 4.9 ± 0.3 ^cde^ | > 50 | > 50 | 16.7 ± 7.2 ^cd^ | > 50 | > 50 |
|  | Succinic | 4.21, 5.64 | -0.59 | ―COOH | > 50 | > 50 | > 50 | > 50 | > 50 | > 50 |
|  | Crotonic | 4.82 | 0.72 | N/A | 0.7 ± 0.2 ^a^ | 10.5 ± 2.5 ^a^ | 31.7 ± 10.0 | 1.3 ± 0.4 ^a^ | 41.7 ± 14.4 ^ab^ | > 50 |
|  | Tartaric | 2.72, 4.34 | -0.76 | ―COOH, ―OH | > 50 | > 50 | > 50 | > 50 | > 50 | > 50 |
|  | Malic | 3.51, 5.03 | -1.26 | ―COOH, ―OH | > 50 | > 50 | > 50 | > 50 | > 50 | > 50 |
|  | Itaconic | 3.65, 5.55 | 0.05 | ―COOH, ―CH_2_ | > 50 | > 50 | > 50 | > 50 | > 50 | > 50 |
| 5 | Valeric | 4.84 | 1.39 | N/A | 3.5 ± 0.8 ^bcd^ | 29.1 ± 0.3 ^abc^ | > 50 | 12.5 ± 0.0 ^bcd^ | > 50 | > 50 |
|  | Citric | 3.13, 4.76, 6.60 | -1.64 | ―COOH, ―OH | > 50 | > 50 | > 50 | > 50 | > 50 | > 50 |
|  | Isocitric | 3.29, 4.71, 6.40 | -1.40 | ―COOH, ―OH | > 50 | > 50 | > 50 | > 50 | > 50 | > 50 |
| 6 | Caproic | 4.88 | 1.92 | N/A | 2.0 ± 0.3 ^ab^ | 12.2 ± 0.3 ^a^ | 31.0 ± 0.8 | 3.6 ± 1.3 ^ab^ | 25 ± 0.0 ^a^ | > 50 |

^a-f^ Different superscript letters indicate significant differences within the column by Tukey’s test (*P* < 0.05). Data are shown as mean ± SD (n ≥ 3). Background colours filled with red and green represent cell (or biofilm) inhibition or not respectively. N/A represents that no additional side groups is attached to the carboxylic acid.

**TABLE S2** Potential of SCCA to eradicate *S. enterica* biofilms. Strains DSM 17058, CCM 3812 and CCM 4420 were cultivated with SCCA to allow biofilm formation and considered as 1^st^ acid treatment (0.78 – 50 mM). The biofilms in microtiter plates were subjected to 2^nd^ acid treatment (0.4 – 50 mM) to determine the minimum biofilm eradication concentrations (MBEC). These experiments were conducted at adjusted pH 4.5, and the MBEC were recorded as the lowest concentration of SCCA when OD_600_ after – OD_600_ before < 0.1 to indicate no growth of biofilm cells.

| **1^st^ acid treatment (mM)** | **Minimum biofilm eradication concentration (mM)** | | | | | | | | |
| --- | --- | --- | --- | --- | --- | --- | --- | --- | --- |
|  | **Formic** | | | **Acetic** | | | **Propionic** | | |
|  | **DSM17058** | **CCM3812** | **CCM4420** | **DSM17058** | **CCM3812** | **CCM4420** | **DSM17058** | **CCM3812** | **CCM4420** |
| 0 | 41.7 ± 14.4 | 37.5 ± 14.4 | 50.0 ± 0.0 | 40.0 ± 13.7 | 43.8 ± 12.5 | 45.0 ± 11.2 | 25.0 ± 0.0 | 22.5 ± 5.6 | 25 ± 0.0 |
| 0.78 | > 50 | 33.3 ± 14.4 | > 50 | 43.8 ± 12.5 | 25.0 ± 0.0 | 41.7 ± 14.4 | 37.5 ± 14.4 | 16.7 ± 7.2 | 20.8 ± 7.2 |
| 1.56 | > 50 | 29.2 ± 19.1 | > 50 | 43.8 ± 12.5 | 8.3 ± 3.6 | 41.7 ± 14.4 | 37.5 ± 14.4 | 10.4 ± 3.6 | 16.7 ± 7.2 |
| 3.13 | > 50 | 14.6 ± 9.5 | 37.5 ± 21.7 | 37.5 ± 14.4 | 3.6 ± 2.4 | 18.8 ± 10.8 | 25.0 ± 0.0 | 4.4 ± 3.2 | 5.2 ± 1.8 |
| 6.25 | > 50 | 6.8 ± 5.5 | 22.9 ± 23.7 | 21.9 ± 6.3 | 1.3 ± 0.5 | 7.3 ± 4.8 | 21.9 ± 6.3 | 1.8 ± 1.2 | 1.7 ± 1.4 |
| 12.5 | 41.7 ± 14.4 | 0.9 ± 0.6 | 1.6 ± 1.6 | 10.9 ± 3.1 | 0.5 ± 0.2 | 1.3 ± 1.6 | 9.4 ± 3.6 | 1.4 ± 1.5 | 0.4 ± 0.0 |
| 25 | 13.0 ± 11.7 | 0.4 ± 0.0 | 2.7 ± 3.1 | 4.3 ± 2.3 | 0.4 ± 0.0 | 0.4 ± 0.0 | 2.3 ± 0.9 | 0.8 ± 0.7 | 0.4 ± 0.0 |
| 50 | 1.7 ± 1.4 | 0.4 ± 0.0 | 0.4 ± 0.0 | 0.8 ± 0.6 | 0.4 ± 0.0 | 0.4 ± 0.0 | 0.9 ± 0.5 | 0.5 ± 0.2 | 0.4 ± 0.0 |
|  | **3-Phenyllactic** | | | **Crotonic** | | | **Caproic** | | |
|  | **DSM17058** | **CCM3812** | **CCM4420** | **DSM17058** | **CCM3812** | **CCM4420** | **DSM17058** | **CCM3812** | **CCM4420** |
| 0 | 21.9 ± 6.3 | 21.9 ± 6.3 | 20.0 ± 6.8 | 20.8 ± 7.2 | 25.0 ± 17.7 | 25.0 ± 0.0 | 22.9 ± 23.7 | 10.4 ± 3.6 | 21.9 ± 18.8 |
| 0.78 | 37.5 ± 14.4 | 20.8 ± 7.2 | 25.0 ± 0.0 | 20.8 ± 7.2 | 5.7 ± 5.9 | 3.1 ± 0.0 | 25.0 ± 21.7 | 8.6 ± 4.7 | 12.5 ± 0.0 |
| 1.56 | 37.5 ± 14.4 | 25.0 ± 0.0 | 25.0 ± 0.0 | 9.4 ± 5.4 | 5.3 ± 6.4 | 0.4 ± 0.0 | 10.4 ± 3.6 | 2.5 ± 1.2 | 8.3 ± 3.6 |
| 3.1 | 37.5 ± 14.4 | 16.7 ± 7.2 | 25.0 ± 0.0 | 3.6 ± 2.4 | 4.6 ± 6.9 | 0.4 ± 0.0 | 7.3 ± 4.8 | 1.1 ± 0.6 | 4.2 ± 1.8 |
| 6.3 | 23.4 ± 19.3 | 4.3 ± 3.4 | 7.3 ± 4.8 | 0.8 ± 0.7 | 2.3 ± 3.4 | 0.4 ± 0.0 | 3.6 ± 2.4 | 0.9 ± 0.5 | 0.8 ± 0.7 |
| 12.5 | 4.8 ± 5.3 | 3.3 ± 2.9 | 5.3 ± 6.4 | 0.4 ± 0.0 | 2.3 ± 3.4 | 0.4 ± 0.0 | 0.8 ± 0.7 | 0.6 ± 0.2 | 0.5 ± 0.2 |
| 25 | 6.3 ± 5.4 | 3.3 ± 2.9 | 0.8 ± 0.7 | 0.4 ± 0.0 | 0.4 ± 0.0 | 0.4 ± 0.0 | 0.4 ± 0.0 | 0.5 ± 0.2 | 0.4 ± 0.0 |
| 50 | 1.2 ± 1.3 | 0.4 ± 0.0 | 0.4 ± 0.0 | 0.4 ± 0.0 | 0.4 ± 0.0 | 0.4 ± 0.0 | 0.4 ± 0.0 | 0.5 ± 0.2 | 0.4 ± 0.0 |

Data are shown as mean ± SD (n ≥ 3). Background colours filled with red and green represent cell inhibition or not respectively.

**TABLE S3** Normality test of the MIC_50_ values, intrinsic and extrinsic parameters of SCCA. A Shapiro-Wilk normality test was performed and the *P* values < 0.05 indicated the data was not normally distributed.

| **Parameter** | | **W** | ***p*-value** |
| --- | --- | --- | --- |
| MIC_50_ | | 0.74649 | < 2.2e–16 |
| MIC_bf_ | | 0.70584 | < 2.2e–16 |
| pH | | 0.63359 | < 2.2e–16 |
| pK_a_ | | 0.84534 | < 2.2e–16 |
| Log Kow | | 0.9393 | 1.336e-11 |
| Carbon chain length | | 0.93546 | 4.891e-12 |
| Additional side groups/bondings | Carboxyl group (―COOH) | 0.64915 | < 2.2e–16 |
|  | Hydroxyl group (―OH) | 0.62846 | < 2.2e–16 |
|  | Methyl group (―CH_3_) | 0.30286 | < 2.2e–16 |
|  | Benzene group (―C_6_H_5_) | 0.34987 | < 2.2e–16 |
|  | Double bond (C=C) | 0.26047 | < 2.2e–16 |
|  | Methylene (=CH_2_) | 0.18712 | < 2.2e–16 |
